# COVID-19 outbreak and acute cholecystitis in a Hub Hospital in Milan: wider indications for percutaneous cholecystostomy

**DOI:** 10.1186/s12893-021-01137-y

**Published:** 2021-04-06

**Authors:** Matteo Barabino, Gaetano Piccolo, Arianna Trizzino, Veronica Fedele, Carlo Ferrari, Vincenzo Nicastro, Andrea Pisani Ceretti, Enrico De Nicola, Nicolò Maria Mariani, Marco Giovenzana, Giovanna Scifo, Massimiliano Mazza, Ruggero Vercelli, Roberto Santambrogio, Carmelo Luigiano, Enrico Opocher

**Affiliations:** 1 Unit of HepatoBilioPancreatic and Digestive Surgery, Department of Health Science, San Paolo Hospital, University of Milan, Via Di Rudinì 8, 20142 Milan, Italy; 2 Department of Diagnostic and Interventional Radiology, San Paolo Hospital, University of Milan, Via Di Rudinì 8, 20142 Milan, Italy; 3 Unit of General Surgery, ASST Fatebenefratelli Sacco, Piazza Principessa Clotilde 3, 20121 Milan, Italy; 4 Unit of Digestive Endoscopy, San Paolo Hospital, University of Milan, Via Di Rudinì 8, 20142 Milan, Italy

**Keywords:** Acute cholecystitis, Percutaneous cholecystostomy, COVID-19, SARS-CoV-2, Bedside, Drainage

## Abstract

**Background:**

COVID-19 pandemic has impacted the Italian National Health Care system at many different levels, causing a complete reorganization of surgical wards. In this context, our study retrospectively analysed the management strategy for patients with acute cholecystitis.

**Methods:**

We analysed all patients admitted to our Emergency Department for acute cholecystitis between February and April 2020 and we graded each case according to 2018 Tokyo Guidelines. All patients were tested for positivity to SARS-CoV-2 and received an initial conservative treatment. We focused on patients submitted to cholecystostomy during the acute phase of pandemic and their subsequent disease evolution.

**Results:**

Thirty-seven patients were admitted for acute cholecystitis (13 grade I, 16 grade II, 8 grade III). According to Tokyo Guidelines (2018), patients were successfully treated with antibiotic only, bedside percutaneous transhepatic gallbladder drainage (PC) and laparoscopic cholecystectomy (LC) in 29.7%, 21.6% and 48.7% of cases respectively. Therapeutic strategy of three out of 8 cases, otherwise fit for surgery, submitted to bedside percutaneous transhepatic gallbladder drainage (37.5%), were directly modified by COVID-19 pandemic: one due to the SARS-CoV-2 positivity, while two others due to unavailability of operating room and intensive care unit for post-operative monitoring respectively. Overall success rate of percutaneous cholecystostomy was of 87.5%. The mean post-procedural hospitalization length was 9 days, and no related adverse events were observed apart from transient parietal bleeding, conservatively treated. Once discharged, two patients required readmission because of acute biliary symptoms. Median time of drainage removal was 43 days and only 50% patients thereafter underwent cholecystectomy.

**Conclusions:**

Percutaneous cholecystostomy has shown to be an effective and safe treatment thus acquiring an increased relevance in the first phase of the pandemic. Nowadays, considering we are forced to live with the SARS-CoV-2 virus, PC should be considered as a virtuous, alternative tool for potentially all COVID-19 positive patients and selectively for negative cases unresponsive to conservative therapy and unfit for surgery.

## Background

COVID-19 is a respiratory tract infection caused by the new SARS-CoV-2 virus, firstly recognized in December 2019 in Wuhan city, Hubei, China.

Globally, on January 2021, there have been 85.929.428 confirmed cases of COVID-19, including 1.876.100 deaths, reported to WHO [1]. The first Italian case has been notified in Codogno (Lodi, Lombardy) on the 21^st^ of February 2020 [2].

After that, the infection rapidly spread throughout Italy and we are currently counting a total of 2.201.945 confirmed cases with 76.877 deaths [1]. Among Italian regions, COVID-19 struck Lombardy the most with 493.022 positive cases, of which 73.069 in the city of Milan [3].During the first two months of the pandemic, from March to April 2020, our Emergency Department (ED) has been quickly re-adapted in order to face the incoming crisis. The majority of human and economic resources were allocated for the symptomatic patients’ care in the ED. The activity of the different surgical specialties was temporarily reorganized by incorporating the urgent and oncologic cases of all surgical branches into one single common ward (30 beds).

The beginning of the pandemic represented a challenge under many aspects, most importantly ensuring the safety for both patients and healthcare workers. Clear instructions on the management of acute surgical disease in COVID-19 patients were initially lacking.

We often deviated from the traditional therapeutic pathway because lack of resources, namely nurses, anaesthesiologists and availability of ICU beds. In this context of general unpreparedness, the secular Hippocrates principle of “*primum non nocere*” became even harder to achieved, as witnessed by other colleagues [4].

Afterward, thanks to a better understanding of the evolution of the global pandemic, we adopted COVID-19 specific guidelines published as time passed by.

In our study, we retrospectively analysed our temporary management strategy for acute cholecystitis (AC) and how it has been influenced by the initial acute phase of COVID-19 pandemic in Italy.

## Methods

### Study design

We retrieved medical records of all patients admitted to our Emergency Department for Acute Cholecystitis (AC) from February 27^th^ to April 30^th^, 2020. We graded the severity of each AC according to the 2018 Tokyo Guidelines (TG-18) [5]. The American Society of Anesthesiologists score (ASA) and the Charlson Comorbidity Index (CCI) were adopted to evaluate the surgical risk [6, 7].

We also estimated the SARS-CoV-2 infection risk by administering a questionnaire regarding COVID-19 symptomatology and possible contacts with infected cases. Moreover, all patients were tested for positivity to SARS-CoV-2 by means of a nasopharyngeal swab.

**Fig. 1:**
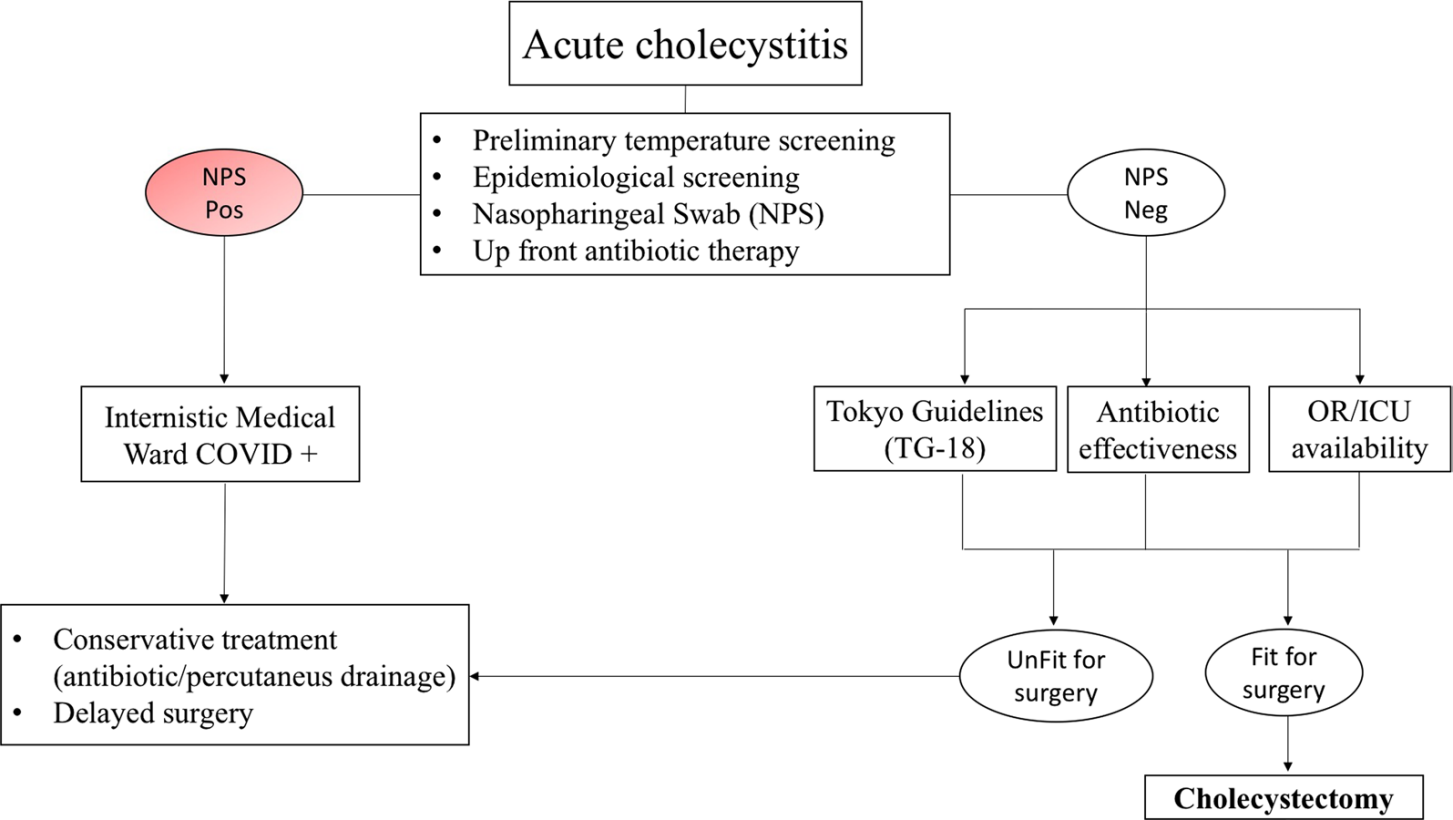
Flowchart of patients affected by acute cholecystitis during the outbreak period of SARS-CoV-2 infection (NPS: nasopharyngeal swab; TG-18: Tokyo Guidelines 2018; OR: operative room; ICU: intensive care unit)

With regards to the standard indications provided by the Tokyo Guidelines, we introduced, due to force majeure, COVID as a variable (Fig. 1): in case of positivity to SARS-CoV-2, patients were admitted to COVID-dedicated wards and they were treated conservatively (antibiotic versus bedside percutaneous drainage). Otherwise, if nasopharyngeal swabs were negative and considering the limited availability of operating theatres and medical staff, we adopted conservative strategy whenever possible, thus reserving surgery for selected patients only. This approach was found to be in line with Italian national surgical protocols and with the American College of Surgeons and the Royal College COVID-19 guidelines [8, 9]. When antibiotic therapy failed, cholecystectomy was recommended, according to the TG-18 [5]. Finally, patients showing no benefits from antibiotic treatment, but considered not suitable for surgery received bedside percutaneous cholecystostomy (PC).

Design, data acquisition, statistical methods and manuscript preparation were carried out according to STROBE guidelines for the strengthening of the reporting of observational studies [10].

### Ethics statement

This study was conducted in accordance with the Declaration of Helsinki (6th revision, 2008) of the World Medical Association. The study was approved by the Institutional Review Committee (IRC) of the Department of Health Sciences, University of Milan (Italy). Patient’s informed consent was not required for this study, due to its retrospective nature.

## Results

### Overall results

During the first phase of COVID-19 pandemic, we observed 37 patients admitted to ED for AC. The mean age of our patients was 64 (range: 38–94 yo) with a slight male prevalence (56.7%). Patients were stratified according to the TG-18: 13 grade I, 16 grade II and 8 grade III. The remaining demographic, clinical and perioperative characteristics of our population are summarized in Table 1.

**Table 1:** Demographic, Clinical and Operative Data of Patients

Age (yo—mean and range)	64 (38–94)
Sex (%)	
Male	21 (56.7%)
Female	16 (43.3%)
BMI^a^ (mean ± SD)	26. 5 (± 3.9)
ASA^b^ score (%)	
1–2	27 (72.9%)
3–4	10 (27.1%)
CCI^c^ (%)	
< 6	32 (86.5%)
≥ 6	5 (13.5%)
Severity grade according to 2018 Tokyo Guidelines (%)	
Grade I	13 (35.1%)
Grade II	16 (43.3%)
Grade III	8 (21.6%)
Treatment (%)	
Antibiotics only	11 (29.7%)
PC^d^	8 (21.6%)
LC^e^	18 (48.6%)

^a^Body Mass Index; ^b^American Society of Anesthesiology;^c^Charlson Comorbidity Index; ^d^ Percutaneous cholecystostomy; ^e^Laparsocopic cholecystectomy

One patient resulted SARS-CoV-2 positive and was admitted to the Internal Medicine COVID ward where he was treated conservatively (antibiotics, fluid resuscitation and bowel rest). Three days after the beginning of antibiotic therapy, the patient developed early clinical features of sepsis and underwent an emergency bedside PC. This procedure improved the clinical picture and the patient was discharged on the tenth day after the nasopharyngeal swab test negativitization.

In SARS-CoV-2 negative patients, a complete resolution of AC was achieved with only antibiotic therapy in 11 out of 36 cases (30.5%). Eighteen patients (50%) with a low surgical risk (ASA 1–2, CCI < 6 and < 75 yo) underwent laparoscopic cholecystectomy (LC) following the initial antibiotic therapy. Among SARS-CoV-2 negative patients, PC was performed in 7 cases (19.4%), of which 1 patient grade I, 3 grade II and 3 grade III, according to TG-18 AC severity grading. Success rate of PC was of 87.5% and the mean post-procedural hospitalization length was 9 days (± 3 days). Only one patient required emergency LC due to a persistent septic status 5 days after the drainage placement. The following post-operative course was regular and the patient was discharged on the third post-operative day (POD). The allocation of different approaches according to AC severity is summarized in Table 2. Of 8 patients receiving a cholecystostomy, one experienced an immediate complication (transient parietal bleeding) requiring conservative treatment (blood transfusion and endovenous infusion of tranexamic acid). Two patients (25%) required readmission after PC due to the development of cholecystitis and cholangitis; they were both treated conservatively.

**Table 2:** Therapeutic approaches according to severity grades (TG-18^a^)

	Grade I	Grade II	Grade III
Antibiotics only	0	7 (43.7%)	4 (50%)
PC^b^	2 (15.4%)	3 (18.7%)	3 (37.5%)
LC^c^	11 (84.6%)	6 (37.6%)	1 (12.5%)

^a^ 2018 Tokyo Guidelines; ^b^ Percutaneous cholecystostomy; ^c^ Laparoscopic cholecystectomy

Average time of drainage removal was 43 days (range 5 to 83), and only half of patients were definitively submitted to LC.

### Results according to TG-18 severity grading

#### Grade I

Almost all patients (11/13) were considered fit for surgery (ASA1-2, CCI < 6 and age < 75 yo) and were admitted to the ED within the optimal timeframe (symptoms onset < 72 h) to be managed by early LC. We did not experience any conversion to laparotomy. Two patients developed post-operative jaundice due to residual common bile duct stones requiring ERCP. One patient had post-operative fever due to a 15 mm fluid collection in the gallbladder bed, successfully treated with antibiotics. In all these cases, the remaining postoperative course was uneventful and all patients recovered successfully. The mean hospital stay was 5 days (range 2–12).

**Fig. 2:**
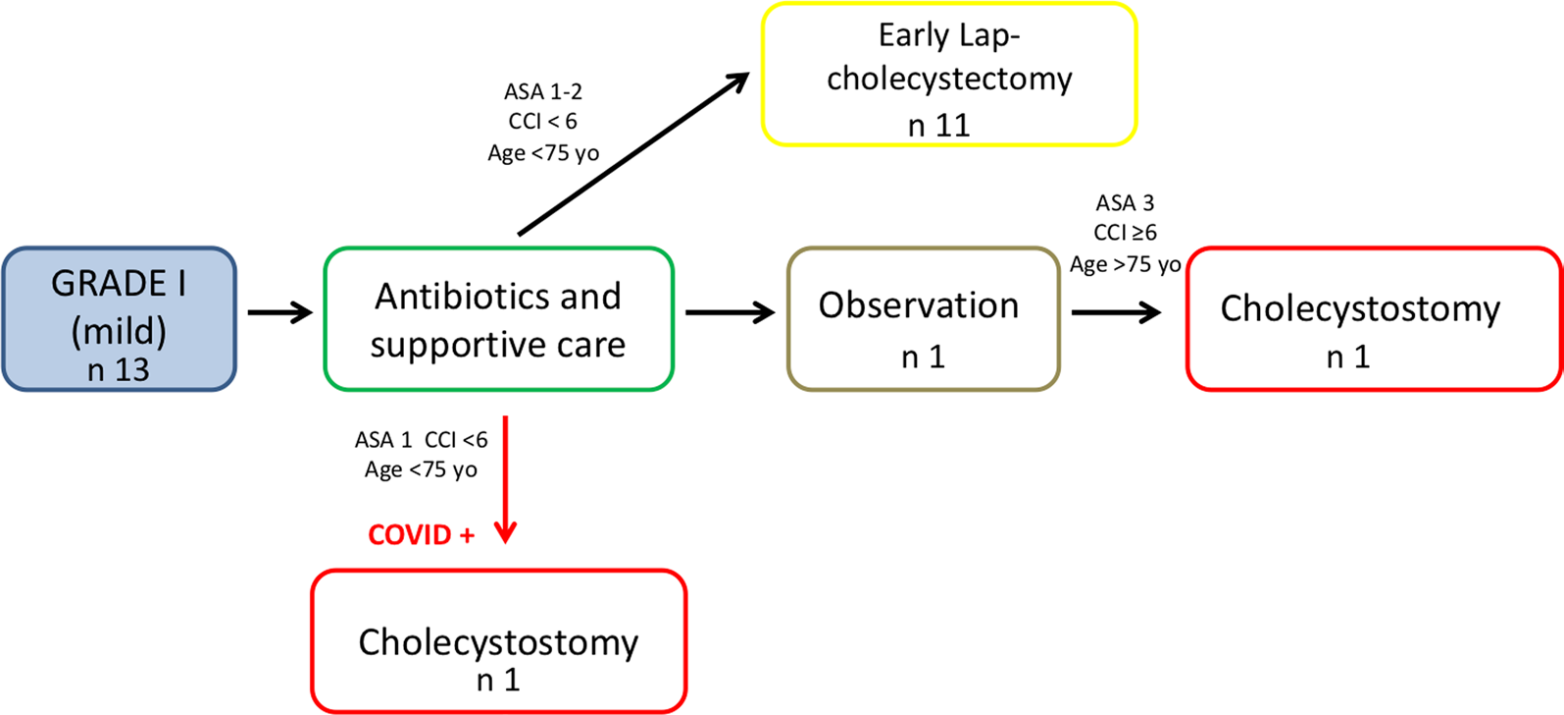
Treatment strategy in Grade I acute cholecystitis

Two cases were treated with PC. One patient with high surgical risk (ASA 3, CCI 6, 88 yo) did not respond to broad spectrum antibiotics and received a bedside PC. The second case is represented by a 58 years-old man, fit for surgery, but SARS-Cov2 positive: therefore, the patient received upfront PC (Fig. 2).

#### Grade II

**Fig. 3:**
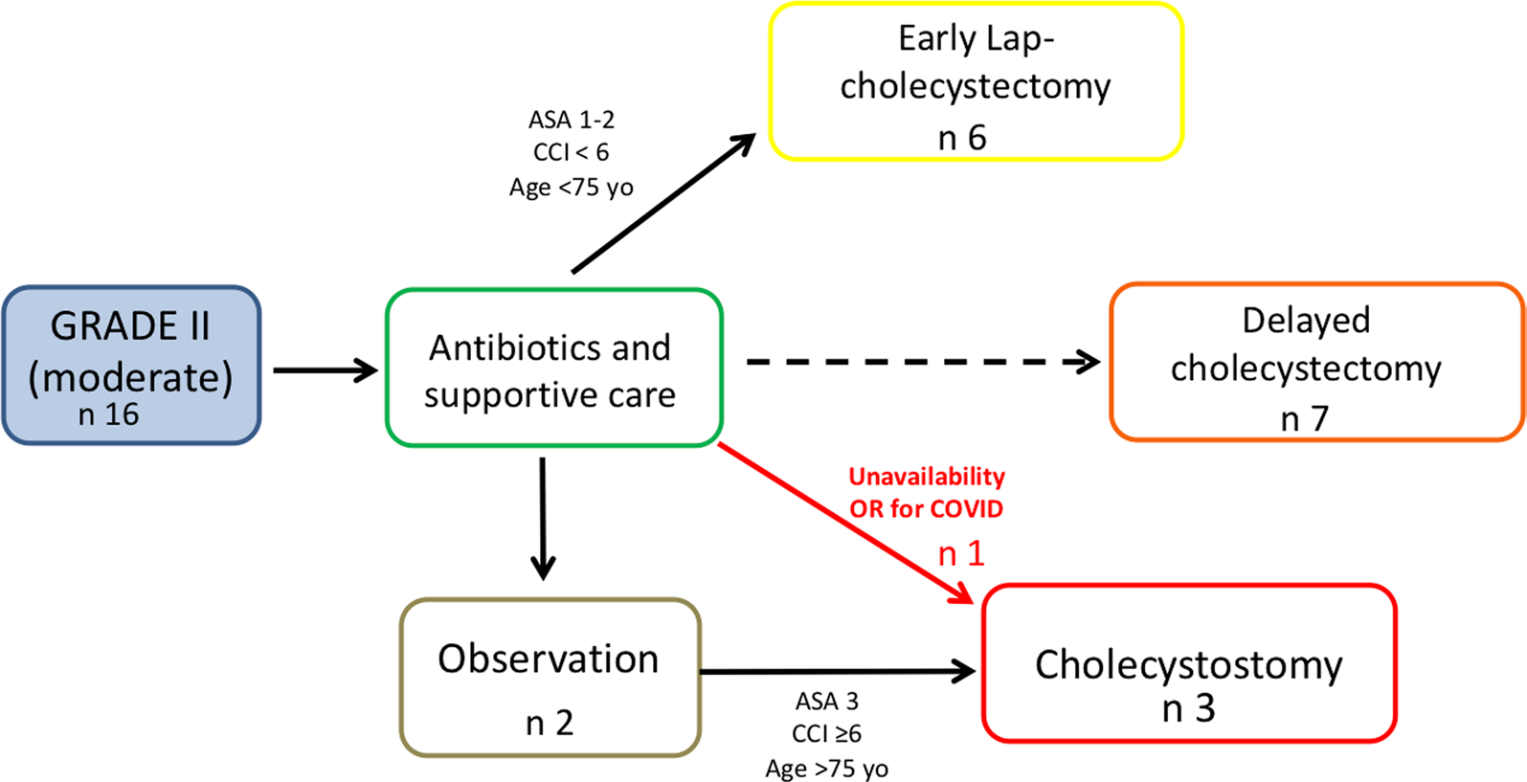
Treatment strategy in Grade II acute cholecystitis

Out of 16 patients, 7 showed a resolution of the clinical picture by means of antibiotic therapy alone. In seven patients with low surgical risk (ASA 1–2, CCI < 6 and age < 75 yo), early LC was indicated: of these, one patient could not be operated due to operating room staff unavailability caused by the COVID emergency. This patient was treated with percutaneous drainage. Two patients did not respond to antibiotic and were also considered not fit for surgery (ASA 3, CCI > 6 and age > 75 yo); therefore, they underwent bedside PC (Fig. 3).

#### Grade III

**Fig. 4:**
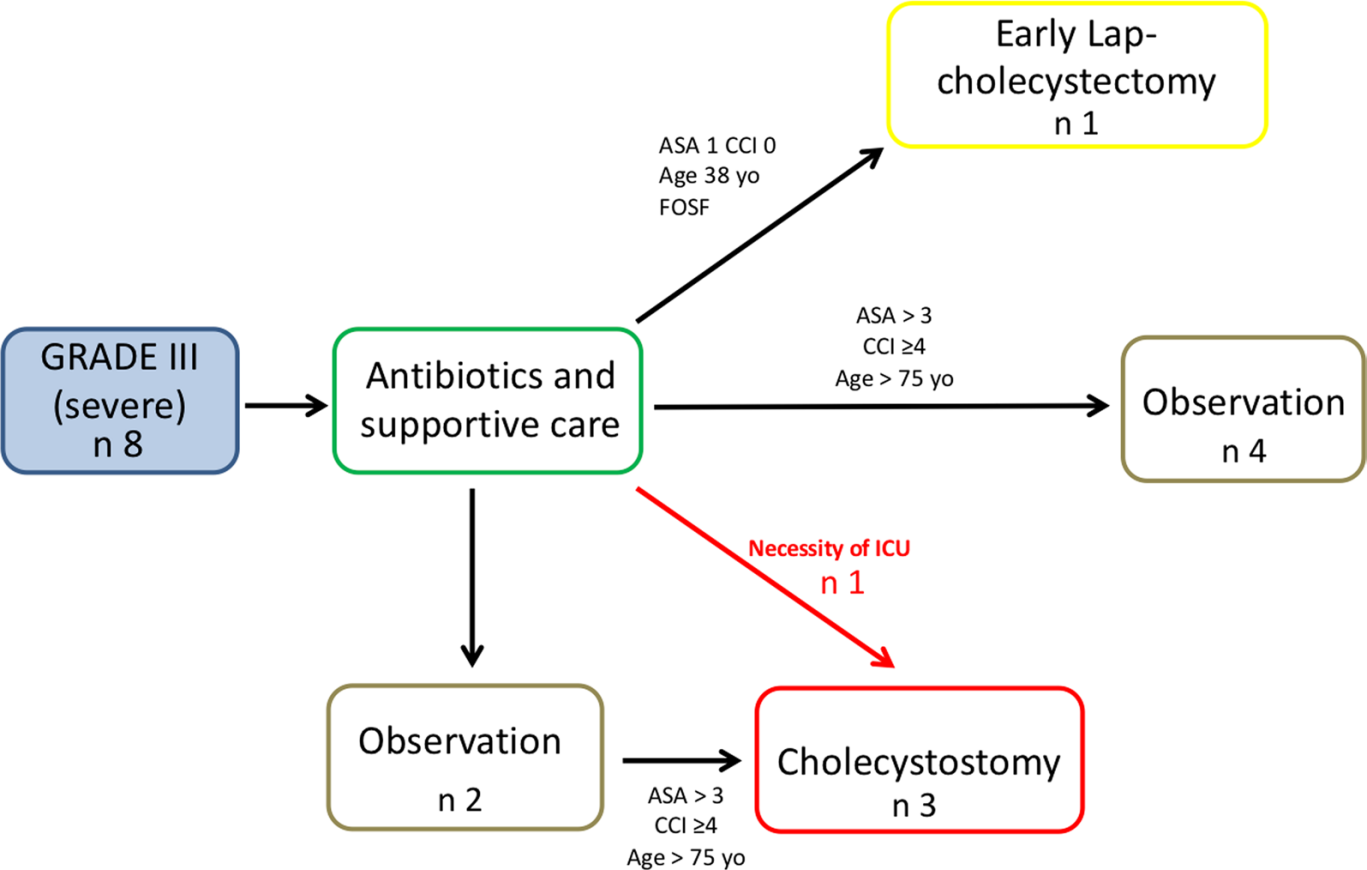
Treatment strategy in Grade III acute cholecystitis

Fifty percent (4/8) of cases showed a resolution of the clinical picture with conservative treatment alone. A young patient with low surgical risk (ASA 1, CCI 0) underwent surgical treatment due to lack of response to antibiotic therapy. Three patients underwent a bedside PC: two elderly patients with many comorbidities (ASA > 3, CCI > 4) and a young, asthmatic female that would have required post-operative ICU monitoring. (Fig. 4).

## Discussion

The first acute phase of SARS-CoV-2 outbreak (from February to April 2020) had a significant impact on elective and emergency surgical care in Italy and especially in Lombardy.

Chinese preliminary data reported that asymptomatic COVID-19-positive patients undergoing surgery early developed pneumonia with increased global mortality rate and unfavorable clinical outcomes [11]. Therefore, most elective surgery has been postponed, especially procedures that would have required intensive care support [12].

Our Surgical Departments were completely reorganized: urgent and oncologic cases of all surgical branches (General, Vascular, Otolaryngology, Maxillofacial Surgery, Urology and Thoracic Surgery) were incorporated in a single surgical ward. This led to competition among different specialists for access to the limited operating rooms. Oncologic patients at high risk of COVID-19 complications (elderly with many comorbidities) were offered neoadjuvant treatments, while definitive surgery was delayed. At first, there was lack of evidence and guideline about the management of the patients admitted in the emergency room (ER) with acute surgical pathologies. On the other hand, several surgical societies have only later released their position statements [8, 9, 13, 14].

The British Intercollegiate General Surgery Guidance (BIGSG) on COVID-19 stated that during the COVID-19 pandemic, whenever non-operative management is possible (such as for early appendicitis and acute cholecystitis), this should be pursued [9].

BIGSG recommended either non-surgical management or the utilization of a percutaneous cholecystostomy tube for the management of acute biliary disease [9].

This new approach clashed on gold standard approach, where early laparoscopic cholecystectomy was recommended, while cholecystostomy was reserved only for those patients considered unfit for surgery [4, 15].

According to this, other surgical societies, such as the Società Italiana di Chirurgia Endoscopica (SICE), Society of American Gastrointestinal and Endoscopic Surgeons (SAGES) and the European Association for Endoscopic Surgery (EAES) have recommended a more patient/hospital-centered and conservative approach [13, 14].

According to those advices, we adopted a paradigm shift towards a non-operative management for some acute surgical pathologies, including acute biliary diseases, in our Surgical Department during the first pandemic phase.

We shifted 3 patients with acute cholecysitits towards a PC instead of urgent laparoscopic cholecystectomy; one case was due to SARS-CoV-2 positivity, and two cases because of unavailability of operative room and post-operative ICU beds. In the other 5 patients, the choice to perform PC fulfilled Tokyo guidelines, regardless pandemic conditioning.

In the following months, a great deal of papers has been published and they now constitute a solid knowledge about the management of surgical patients in the COVID-19 era [16–22].

The most important findings concerned mortality and pulmonary complications in patients undergoing surgery with pre- or peri-operative SARS-CoV-2 infection [16, 19].

In an Italian matched cohort study, surgical mortality and complications were higher in patients with COVID-19 compared with patients without coronavirus infection [16].

A large international cohort study (COVIDSurg Collaborative Group) proved that pulmonary complications occurred in half of COVID-19 patients with higher mortality, especially in men aged 70 years and older [19]. These data suggest also that there was an increased postoperative mortality in COVID-19 patients even if the infection was acquired in the postoperative period [16, 19].

Therefore, we’ve strengthened ourselves that PC continues to be the best therapeutic strategy for acute cholecystitis in COVID-19 positive patients during all the pandemic.

On the other hand, we do not consider PC as an “a priori” choice in SARS-CoV-2 negative patients otherwise fit for surgery.

In several studies, the SARS-CoV-2 RNA has been detected in the peritoneal cavity [23, 24], however there is no evidence that indicates the presence of SARS-CoV-2 in surgical smoke [25], therefore the potential risk of virus transmission to the healthcare staff during laparoscopy has not yet been confirmed.

Moreover, there are no data reporting higher rates of COVID-19 infection related to laparoscopic cholecystectomy with respect to the open approach. 

For this reason, we think that patients should not be denied the benefits of laparoscopy in terms of mortality, morbidity, and postoperative hospital stay also during COVID-19 period [26–28].

We adopted a standard laparoscopic approach and, in order to limit any possible spread of the virus due to nebulization during laparoscopy, the surgical team was provided with adequate personal protective equipment (FFP2 masks and visors). We also implemented a specific filtration system for a safe carbon dioxide evacuation at the end of the laparoscopic procedure. The filtration system is composed of a small rubber tube connecting the gas outlet of one of the trocars to a filter usually mounted on mechanical ventilation machines (Minz’s device) [29].

Anyway, in SARS-CoV-2 negative patients unfit for surgery, percutaneous drainage of the gallbladder remained a safe and often temporary effective (success rate of 85%) [30] option after failure of conservative antibiotic therapy.

Among the various techniques such as transpapillary, transmural and percutaneous transhepatic drainage of the gallbladder, the latter is the more frequently chosen due to its lower risk of bile leakage, simplicity of execution and reproducibility at the patient’s bedside [31].

In literature the correct timing for performing PC is still debated and the scientific community is divided between the early (within 24 h from the admission) and the late (after 24 h from the admission) approach [32–34].

When the procedure is performed within the first 24 h following the onset of symptoms, gallbladder drainage is related to shorter hospital stay and low complication rate (0.5%), especially bleeding [31].

In our experience, we performed cholecystostomy in all patients within 72 h from the onset of clinical signs and we did not observe any peri-procedural complication apart from one case of transient parietal bleeding.

The Rose Surgical Collaborative UK retrospective cohort study on 864 patients with a diagnosis of cholecystitis shows that 22 out of 63 patients (35,2%) undergoing cholecystostomy experienced a complication of which 4,7% were immediate while 85,3% occurred later [35].

Wrong site PC placement and displacement was reported respectively in 2 (10,5%) and 12 (63,2%) cases [35]. Moreover, Lei et al. reported a drainage occlusion rate of 10% [36].

Cholecystostomy drain management is not standardized yet. Some authors suggest a check tubogram before discharge [35], but it could represent a trigger for cholangitis/cholecystitis relapse before elective surgery. In the series published by Lu et al. 42,9% of patients undergoing PC required readmission to hospital for relapsing biliary symptoms (range 1 to 4 times each) [35].

In our experience, we obtained the complete remission of symptoms within 24 h after percutaneous drainage in 87.5% of cases. Only one patient underwent urgent LC due to a persistent septic status 5 days after PC. Cholecystostomy drain remained in place for a median time of 43 days without any routine imaging check before its removal. In 2 cases a readmission was needed to manage symptoms relapse, thus conditioning extended time before scheduled LC.

Our study has some limitations: the relatively small sample size, the retrospective nature and the single-center involvement. Despite the shortness of cases, this study wants to draw a picture of an ongoing view of strategy adopted along the actual pandemic in one of the first and most affected country in the world.

## Conclusions

Percutaneous gallbladder drainage has shown to be an effective and safe treatment thus acquiring an increased relevance during SARS-CoV-2 outbreak. Indications to PC became more selective according to the better understanding of the ongoing pandemic. Cholecystostomy should be most frequently pursued in SARS-CoV-2 positive patients while only in selected case for negative patients, especially in those unresponsive to conservative therapy and unfit for surgery.

## Data Availability

The dataset used during the current study are available at https://www.synapse.org/#!Synapse:syn22162934/files/

## Abbreviations

AC: Acute cholecystitis
ASA: American Society of Anesthesiologists
CCI: Charlson Comorbidity Index
ED: Emergency department
ICU: Intensive care unit
LC: Laparoscopic cholecystectomy
PC: Percutaneous cholecystostomy
SD: Standard deviation
TG-18: 2018 Tokyo guidelines
